# Five New Hypocrealean Species from Algae and Sediment in the Intertidal Zones of China

**DOI:** 10.3390/jof11070476

**Published:** 2025-06-23

**Authors:** Meng-Meng Wang, Wang-Ying Mo, Meng-Yi Sun, Ye-Hui Tu, Wei Li

**Affiliations:** 1 Guangdong Provincial Key Laboratory of Marine Biotechnology, Shantou University, Shantou 515000, China; mmwang@stu.edu.cn (M.-M.W.); 23wymo@stu.edu.cn (W.-Y.M.); 23mysun@stu.edu.cn (M.-Y.S.); 22yhtu1@stu.edu.cn (Y.-H.T.); 2 Guangdong Provincial Key Laboratory of Marine Disaster Prediction and Prevention, Shantou University, Shantou 515000, China; 3 Shantou Key Laboratory of Marine Microbial Resources and Interactions with Environment, Shantou University, Shantou 515000, China

**Keywords:** marine fungi, *Hypocreales*, taxonomy, Chinese seas

## Abstract

Hypocrealean fungi are a well-documented group of fungi, with a wide range of ecological roles that include saprobic, parasitic, and endophytic forms, capable of thriving in diverse environments, both terrestrial and marine. Members in this group are abundant and widely distributed in marine environments around the world. However, the species diversity and distribution of this fungal group in Chinese seas is rarely reported. This study introduces five new species, namely *Fusarium flavoides* M.M. Wang & W. Li, *Gliomastix fasciculata* M.M. Wang & W. Li, *Marquandomyces ulvae* M.M. Wang & W. Li, *Stephanonectria arenicola* M.M. Wang & W. Li, and *Verruciconidia oligospora* M.M. Wang & W. Li, based on morphological characteristics and LSU-ITS-tef1-rpb2 phylogenetics. These new species were discovered from marine algae (*Ulva spinulosa*) and sediments (mud and sand). A detailed comparison of these new species and their close relatives is also presented.

## 1. Introduction

The order *Hypocreales* Lindau was established in 1897, and recently this fungal group is now considered to be one of the most substantial orders of the class *Sordariomycetes* [1], comprising approximately 320 genera in 15 families [2]. Historically, the classification of this order has undergone numerous revisions, with a modified characterization of the Hypocreales being proposed in 1970 [3]. Subsequent to this, a considerable number of species were assigned to this order, and an updated taxonomic system for this group of fungi was conducted. Morphologically, members of this order, also known as hypocrealean fungi, are frequently characterized by colorful sexual structures such as white, light orange, and black, and the typically asexual structures that were summarized as *Acremonium*-like, *Cylindrocarpon*-like, *Fusarium*-like, *Paecilomyces*-like, and so on [4,5,6,7].

Hypocrealean fungi exhibit a broad spectrum of host/habitat and ecological functions, encompassing saprophytes, parasites, and endophytes in soil and plants in terrestrial habitats [7,8]. Furthermore, these fungi have been identified in a wide range of marine environments around the world. For instance, studies using culturable methods have revealed that species in genera *Acremonium* Link and *Fusarium* Link have a worldwide distribution in sediments, corals, sponges, sea fans, and seaweeds [9,10,11,12,13]. However, the distribution of culturable hypocrealean fungi in Chinese seas has been sporadically reported, with the exception of *Acremonium egyptiacum* (J.F.H. Beyma) W. Gams, *Emericellopsis maritima* Beliakova, and *Neocosmospora solani* (Mart.) L. Lombard & Crous from marine carbs in Taiwan [14], *Fusarium aseptatum* Meng Li & L. Cai from mangrove sediment in Guangdong [15], and several species including *A. egyptiacum* and *Purpureocillium lilacinum* (Thom) Luangsa-ard, Houbraken, Hywel-Jones & Samson from marine algae in Shandong [16]. Considering the high relative abundances of these fungi revealed by unculturable methods in the Yellow Sea of China [17] and their ecological roles (endophyte, parasite, pathogen, and saprotroph) identified by FungalTraits [18], we believe that the marine environments in China should be inhabited by diverse hypocrealean fungi that probably perform important ecological functions.

During the past decade, our group has conducted a long-term survey of fungal diversity in Chinese seas (e.g., [19,20,21,22]) and accumulated a number of hypocrealean strains from various marine samples such as algae, seawater, and sediment. The present study introduced five new species from marine algae and sediments in the intertidal zones, based on their morphology and culture characteristics. The taxonomic placements of these new species were confirmed by multi-locus phylogenetic analyses of LSU, ITS, tef1, and rpb2. Comparisons were made between the new species and their close relatives.

## 2. Materials and Methods

### 2.1. Sample Collection and Fungal Isolation

Algae samples were collected from intertidal zones in the Guangdong province (Shenzhen), while sediment samples (2–20 cm depth) were obtained from the Liaoning province (Dalian and Huludao) and the Shandong province (Qingdao, Weihai and Yantai). The collection of samples was conducted in accordance with the protocol described in Wang et al. [22]. The isolation of fungal strains from the algae and sediment samples was undertaken employing the protocol outlined by Wang et al. [22]. In summary, samples of algae and sediment were collected and stored in sterile bags in a 4 °C freezer and transferred to the laboratory promptly. The algae samples were then cut into 0.5 × (0.2–0.5) cm segments, surface-sterilised, and placed onto isolation media (tissue-isolation method). Segments were homogenised, diluted to a series of concentrations (10^−1^, 10^−2^, 10^−3^, and 10^−4^), and spread onto isolation media (dilution plate method). Sediment samples were directly dispersed onto isolated medium plates (direct isolation method), and suspended, cultivated by shaking, diluted (10^−1^, 10^−2^, 10^−3^, and 10^−4^), and plated onto isolation media (dilution plate method) [22]. Fifty types of media that are consistent with the formula of Wang et al. [22], including Martin medium (MM), 1/10 potato dextrose agar (1/10 PDA), 1/5 malt extract agar (1/5MEA), corn meal agar (CMA), and yeast extract peptone glucose agar (YPG), were used as isolation media in this study. Fungal isolates were picked up with a sterilised needle and transferred onto potato dextrose agar (PDA) plates when individual colonies were observed. All isolates examined in this study were deposited in Wei Li’s personal culture collection (WL). Type specimens of new species were deposited in the Fungarium of the Institute of Microbiology (HMAS; https://nmdc.cn/fungarium/, accessed on 15 January 2025), with the ex-type living cultures in the China General Microbiological Culture Collection Center (CGMCC; https://www.cgmcc.net/, accessed on 15 January 2025).

### 2.2. Morphological Observation

Considering that fungi often exhibit distinct colony morphological characteristics on different culture media, this study employed three different culture media to obtain a more comprehensive understanding of the cultural characteristics of the studied isolates. The isolates studied were incubated in the dark on PDA and oatmeal agar (OA) at 25 °C in the dark for seven days. Seven days later, culture characteristics including colony morphology, pigmentation, and odour were observed. Colours were assessed according to the colour charts of Kornerup and Wanscher [23]. Micromorphological characteristics were examined and photo-documented using water as a mounting medium under an Olympus BX53 microscope with differential interference contrast (DIC) optics [22]. For each species, 30 conidiophores, 30 conidiogenous cells, and 50 conidia were mounted and measured randomly, respectively [22].

### 2.3. DNA Extraction, PCR Amplification and Sequencing

Genomic DNA was extracted from fungal mycelia grown on PDA after 7 days, using a modified CTAB protocol as described in Guo et al. [24]. Briefly, the process of fungal DNA extraction involved the grinding of fresh mycelia with CTAB buffer [24] and quartz sand, followed by incubation at 60 °C for 30 min. Subsequently, 500 μL of a solution of phenol–chloroform (1:1) was added, mixed thoroughly, and subjected to centrifugation (11,900× *g*, 15 min). The aqueous phase was transferred, mixed with chloroform–isoamyl alcohol (24:1) and centrifuged again. The DNA was precipitated using 50 µL of 5M KOAc and 400 µL of isopropanol, mixed gently, centrifuged (9200× *g*, 2 min), washed twice with 70% ethanol, air-dried, and finally resuspended in 100 µL of TE buffer (10 mM Tris-HCl, 1 mM EDTA).

Four loci, including partial large subunit ribosomal RNA gene (LSU), 5.8S nuclear ribosomal RNA gene with the two flanking internal transcribed spacer (ITS) regions, partial translation elongation factor gene (tef1) and partial DNA-directed RNA polymerase II second largest subunit gene (rpb2), were amplified with the primer pairs ITS5 (5′-GGAAGTAAAAGTCGTAACAAGG-3′)/ITS4 (5′-TCCTCCGCTTATTGATATGC-3′) [25], LR0R (5′-ACCCGCTGAACTTAAGC-3′)/LR5 (5′-TCCTGAGGGAAACTTCG-3′) [26,27], EF-983F (5′-GCYCCYGGHCAYCGTGAYTTYAT-3′)/EF2218R (5′-ATGACACCRACRGCRACRGTYTG-3′) [28] and RPB2-5F2 (5′-GGGGWGAYCAGAAGAAGGC-3′)/RPB2-7cR (5′-CCCATRGCTTGYTTRCCCAT-3′) [29,30], respectively. The PCR amplifications were performed in a total volume of 30 μL containing 15 μL of 2 × Rapid Taq Plus Master Mix (Dye Plus) (Vazyme, Nanjing, China), 1 μL of each primer (0.1 μM), 1 μL of genomic DNA (1–10 ng), and 12 μL of ddH2O. The PCR amplifications of LSU, ITS, and tef1 were set as follows: an initial denaturation at 95 °C for 5 min, followed by 35 cycles of denaturation at 95 °C for 1 min, annealing at 48 °C (LSU), 52 °C (ITS), and 56 °C (tef1), respectively, for 1 min, and extension at 72 °C for 1 min, and a final extension step at 72 °C for 10 min. The PCR amplification for rpb2 was set as described by Wang et al. [31]. PCR products for four loci were purified and sequenced in both directions using an ABI DNA analyzer by SinoGenoMax company (Beijing, China). Consensus sequences were obtained using SeqMan of the Lasergene software package v. 14.1 (DNAstar, Madison, WI, USA).

### 2.4. Evolutionary Lineage Modeling

The sequences of the hypocrealean strains examined in this study and the reference strains are listed in Table 1. For each locus, sequences were aligned using ClustalX 2.1 [32], and the alignments were manually adjusted where necessary. The best-fitting nucleotide-substitution modes according to the Akaike Information Criterion (AIC) were selected using mrmodeltest2 [33]. Alignments derived from this study were deposited in TreeBASE (submission ID 31992), and taxonomic novelties were deposited in Fungal Names (https://nmdc.cn/fungalnames/, accessed on 15 January 2025).

Phylogenetic analyses of the combined dataset were performed using Bayesian inference (BI) and maximum-likelihood (ML) methods. The BI analyses were conducted using MrBayes v. 3.2.1 following the protocol of Wang et al. [31], with optimization of each locus treated as a partition in combined analyses, based on the Markov Chain Monte Carlo (MCMC) approach [34]. All characters were equally weighted, and gaps were treated as missing data. The stationarity of the analyses was determined by examining the standard deviation of split frequencies (<0.01) and –ln likelihood plots in AWTY [35]. The ML analyses were conducted using PhyML v. 3.0 [36], with 1000 bootstrap replicates. The general time-reversible model was applied with an invariable gamma-distribution rate variation (GTR+I+G).

## 3. Results

### 3.1. Phylogenetic Analyses

**Figure 1:**
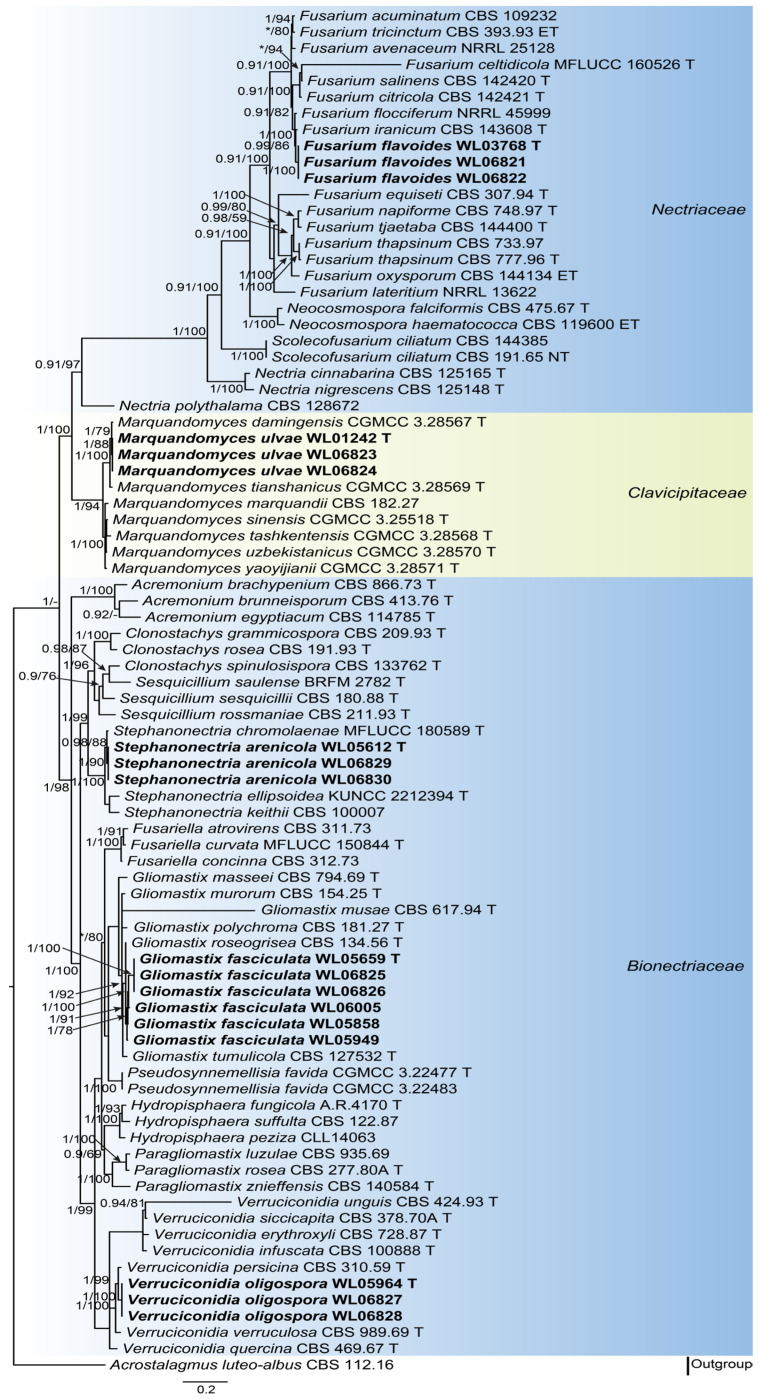
Fifty percent majority rule consensus tree from a Bayesian analysis based on a four-locus combined dataset (LSU-ITS-tef1-rpb2) showing the phylogenetic relationships of these hypocrealean fungi. The Bayesian posterior probabilities (PP > 0.9) and PhyML bootstrap support values (BS > 50%) are displayed at the nodes (PP/BS). Nodes with PP value below 0.9 are displayed as “*”. The tree was rooted to *Acrostalagmus luteo-albus* CBS 112.16. Ex-type cultures are indicated with “T”, epi-type with “ET” and neo-type with “NT”. New species introduced in this paper are marked in bold.

Analyses of the hypocrealean fungal phylogeny were conducted by using a combined LSU (875 bp), ITS (634 bp), tef1 (849 bp) and rpb2 (908 bp) dataset. For the BI analysis, the GTR+I+G model was selected for the LSU, ITS and rpb2 loci, while the GTR+G model was used for the tef1 locus. The phylogeny showed that our five new species were clustered into five genera in three families (*Bionectriaceae* Samuels & Rossman, *Clavicipitaceae* (Lindau) Earle, and *Nectriaceae* Tul. & C. Tul.), namely *Fusarium flavoides*, *Gliomastix fasciculata*, *Marquandomyces ulvae*, *Stephanonectria arenicola,* and *Verruciconidia oligospora* (Figure 1). In order to provide a more accurate illustration of the genetic relationships between the five new species and other species within the same genus, phylogenetic analyses were conducted for each genus based on multi-locus datasets (Supplementary Figure S1).

### 3.2. Taxonomy of the Novel Species

**Figure 2:**
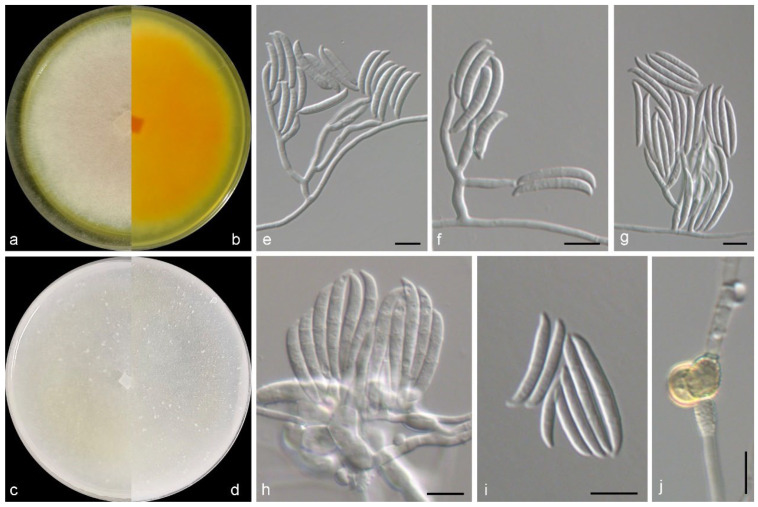
Morphological characters of *Fusarium flavoides* (from ex-type WL03768). (**a**–**d**) Colonies on PDA and OA after 7 d; (**e**–**h**) conidiophores and conidiogenous cells; (**i**) conidia; (**j**) chlamydospores. Bars: (**e**–**j**) = 10 μm.

***Fusarium* *flavoides*** M.M. Wang & W. Li sp. nov., Figure 2.

*Classification: Ascomycota*, *Sordariomycetes*, *Hypocreales*, *Nectriaceae*, *Fusarium*

*FungalNames*: FN 572360

*Etymology*: Epithet refers to the cultural characteristics that yellow pigmentation produced on PDA, “*flav*-” (yellow) + “-*oides*” (similar to) = *flavoides*.

*Typus*: China, Shandong Province, Yantai city (120.70° E, 37.81° N), from intertidal sediment of a sand beach, June 2014, X.M. Bian (HMAS 353470, holotype designated here, dried culture on PDA; culture ex-type CGMCC 3.28711 = WL03768).

Mycelium consisting of branched, septate, hyaline, smooth-, thin-walled hyphae, 2–3 μm wide. Conidiophores mostly aggregated, erect, straight, arising directly from aerial or substratal mycelium, branched, 30–60 μm long, hyaline, smooth-walled, with cell walls usually thicker than those of vegetative hyphae. Phialides solitary, lateral, terminal, subcylindrical, hyaline, thick-, smooth-walled, 7.0–20.5 × 2.5–4.5 μm, commonly with conspicuous periclinal thickening at conidiogenous loci; polyphialides not observed. Macroconidia 2–3-septate, falciform, with a pointed to blunt apical cell and a poorly developed to well-developed foot-shaped basal cell, curved, hyaline, thin-, smooth-walled, eguttulate, 20.0–36.5 × 2.5–5.7 μm; microconidia not observed. Chlamydospores 0–1-septate, subglobose, hyaline and smooth in young and becoming golden and verrucous with maturity, 8.3–9.7 × 6.2–9 μm. Sexual morph not observed.

*Culture characteristics*—Colonies on OA at 25 °C after 7 days reaching 40–45 diam, flat, villose, sparse, colony margin erose, surface white in the centre, yellowish white (2A2) at the margin; reverse white in the centre, yellowish white (2A2) at the margin. Colonies on PDA at 25 °C after 7 days reaching 60–65 mm diam, flat, felty, colony margin filamentous, surface reddish white (9A2) in the centre, white at the margin; reverse light orange (5A5) in the centre, white at the margin, with light yellow (3A4) pigmentation.

*Other examined isolates*: China, Shandong Province, Yantai city (120.70° E, 37.81° N), from intertidal sediment of a sand beach, June 2014, X.M. Bian (WL06821); ibid., WL06822.

*Notes*: In this study, a new *Fusarium* species, namely *F. flavoides* from intertidal sediment, was introduced. Phylogenetically, this species was closely related to *F. flocciferum* Corda and *F. iranicum* Torbati, Arzanlou & Sand.-Den., but differs by 71 bp and 81 bp in the LSU-ITS-tef1-rpb2 dataset, respectively (Figure 1). Morphologically, the above species could be distinguished in the septation, shape and size of macroconidia (2–3-septate, falciform, with a pointed to blunt apical cell and a foot-shaped basal cell, 20–36.5 × 2.5–5.7 μm in *F. flavoides* vs. 3–5-septate, falcate, with tapering apical cells and distinctly pedicellate basal cells, 16–60 × 3–4 μm in *F. flocciferum*, and 0–5-septate, falcate, 15.5−20 × 3–4.5 μm in *F. iranicum*) [37,38,39,40]. These species differ in locations and hosts/habitats: *F. flavoides* was discovered from intertidal sediment in China, while *F. flocciferum* and *F. iranicum* was mostly from soil, plants, and other fungi in Germany, Iran, Netherlands, and other countries [8,41,42]. The new species is similar to *F. thapsinum* Klittich, J.F. Leslie, P.E. Nelson & Marasas in the production of yellowish pigment, but differs in the absence of microconidia (present in *F. thapsinum*) and septation and size of macroconidia (3–8-septate, 24–64 × 3–4 μm in *F. thapsinum*) [43]. Moreover, *F. flavoides* is far from *F. thapsinum* in the LSU-ITS-tef1-rpb2 phylogeny (Figure 1).

**Figure 3:**
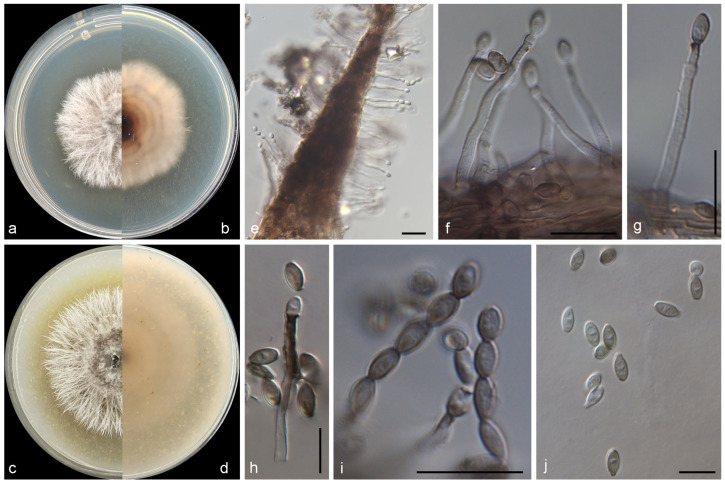
Morphological characters of *Gliomastix fasciculata* (from ex-type WL05659). (**a**–**d**) Colonies on PDA and OA after 7 d; (**e**–**h**) conidiophores and conidiogenous cells; (**i**) conidia in chains; (**j**) conidia. Bars: (**e**–**j**) = 10 μm.

***Gliomastix fasciculata*** M.M. Wang & W. Li sp. nov., Figure 3.

*Classification: Ascomycota*, *Sordariomycetes*, *Hypocreales*, *Bionectriaceae*, *Gliomastix*

*FungalNames*: FN 572361

*Etymology*: Epithet refers to the fascicular conidiophores of this species, “*fasciculata*” in Latin means “fascicular”.

*Typus*: China, Shandong Province, Weihai city (122.18° E, 37.50° N), from intertidal sediment of a sand beach, November 2020, M.M. Wang and Y. Zheng (HMAS 353471, holotype designated here, dried culture on PDA; culture ex-type CGMCC 3.28712 = WL05659).

Mycelium consisting of septate, hyaline, smooth-, thin-walled hyphae, up to 2.5 μm wide when young, becoming thick-walled, up to 3.5 μm wide in old cultures. Conidiophores solitary or aggregated, erect, straight or slightly flexuose at base, arising directly from superficial hyphae, unbranched, 1-septate at base, hyaline, becoming dark brown at the top with age, slightly rough-walled. Phialides lateral, terminal, subulate, 20.0–31.0 × 1.5–3.5 μm wide at base, hyaline at first, dark brown in old cultures, thick-, slightly rough-walled, with conspicuous periclinal thickening and dark brown flared collarettes; polyphialides not observed. Conidia aseptate, ovoid to ellipsoid with truncate, hyaline, thin-walled at beginning, brown to olivaceous brown with age, thick-, smooth-walled in old cultures, 2.8–4.7 × 2.0–3.1 μm, guttulate, arranged in long dry chains. Chlamydospores and sexual morph not observed.

*Culture characteristics*—Colonies on OA at 25 °C after 7 days reaching 40–45 diam, filamentous, crateriform, dusty, colony margin filamentous, surface olive grey (3D1) in the centre, white at the margin; reverse brownish orange (6C4) in the centre, white at the margin, with greyish brown (6D3) pigmentation. Colonies on PDA at 25 °C after 7 days reaching 40–45 mm diam, filamentous, crateriform, dusty, colony margin filamentous, surface olive grey (3D1) in the centre, white at the margin; reverse cognac (6E7) in the centre, white at the margin.

*Other examined isolates*: China, Shandong Province, Weihai city (122.18° E, 37.50° N), from intertidal sediment of a sand beach, November 2020, M.M. Wang and Y. Zheng (WL06825); ibid., WL06826; ibid., Qingdao city (120.47° E, 36.09° N), from intertidal sediment of a sand beach, March 2021, Y. Zheng, Z.H. Pan and Y.R. Ma (WL05858); Liaoning Province, Dalian city, from intertidal sediment of sand beach, May 2021, Y. Zheng and Z.H. Pan (WL05949); ibid., Huludao city (120.80° E, 40.49° N), from intertidal sediment of a sand beach, Y. Zheng and Z.H. Pan (WL06005).

*Notes*: Phylogenetically, the newly introduced species was closely related to *G. polychroma* (J.F.H. Beyma) Matsush., *G. roseogrisea* (S.B. Saksena) Summerb., and *G. tumulicola* (Kiyuna, An, Kigawa & Sugiy.) Summerb. (Figure 1), but differs by 176 bp, 92 bp and 148 bp in the LSU-ITS-tef1-rpb2 dataset, respectively. Morphologically, the four species distinct in the shape, size and pigmentation of conidia (ovoid to ellipsoid, 2.8–4.7 × 2–3.1 μm, brown to olivaceous brown with age in *G. fasciculata*, vs. ellipsoid, oviform, 3–7.5 × 2–4 μm in *G. polychroma*, mostly tear-shaped, 4.9–6.5 × 2.6–4.1 μm, greyish-black in *G. roseogrisea*, 4–5 × 2–3 μm, blackish brown in *G. tumulicola*) [44,45,46]. The above species differ in the hosts/habitats and geographic distribution in that *G. fasciculata* was retrieved from intertidal sediments in China, while *G. polychroma*, *G. roseogrisea,* and *G. tumulicola* from *Hevea brasiliensis*, painting, and soil in India, Indonesia, and Japan [44,45,46].

**Figure 4:**
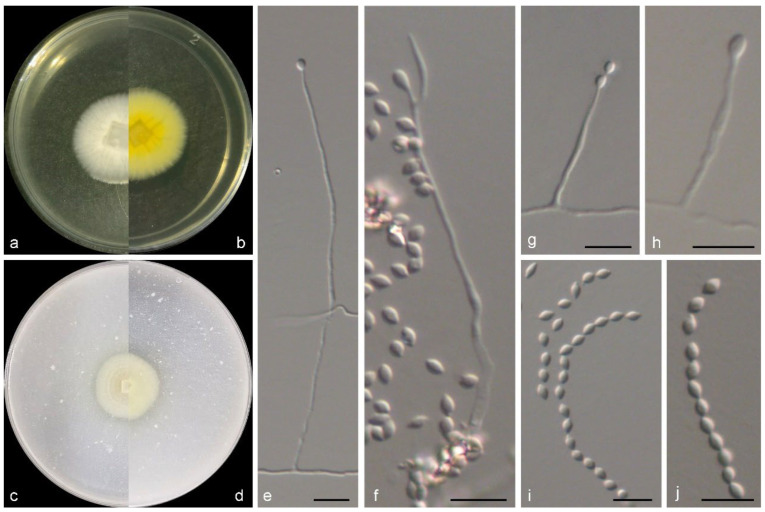
Morphological characters of *Marquandomyces ulvae* (from ex-type WL01242). (**a**–**d**) Colonies on PDA and OA after 7 d; (**e**–**h**) conidiophores and conidiogenous cells; (**i**,**j**) conidia in chains. Bars: (**e**–**j**) = 10 μm.

***Marquandomyces ulvae*** M.M. Wang & W. Li sp. nov., Figure 4.

*Classification: Ascomycota*, *Sordariomycetes*, *Hypocreales*, *Clavicipitaceae*, *Marquandomyces*

*FungalNames*: FN 572362

*Etymology*: Epithet refers to the host genus of the type specimen, *Ulva*.

*Typus*: China, Guangdong Province, Shenzhen city, from marine algae *Ulva spinulosa*, May 2014, M.M. Wang (HMAS 353472, holotype designated here, dried culture on PDA; culture ex-type CGMCC 3.28716 = WL01242).

Mycelium consisting of branched, septate, hyaline, rough-, thin-walled hyphae, up to 3 μm wide. Conidiophores solitary, hyaline, erect, arising from superficial hyphae, unbranched or poorly branched, 15–100 μm long, 2–2.5 μm wide at base. Phialides terminal or lateral, cylindrical or subulate, hyaline, thin-, smooth-walled, 15.0–35.0 × 2.0–2.5 μm wide at base; polyphialides not observed. Conidia aseptate, oval with sharp ends, hyaline, thin-, smooth-walled, 2.5–5 × 2–3.3 μm, arranged in dry, long chains. Chlamydospores and sexual morph not observed.

*Culture characteristics*—Colonies on OA at 25 °C after 7 days reaching 10–12 diam, circular, flat, colony margin erose, surface white; reverse white. Colonies on PDA at 25 °C after 7 days reaching 10–15 mm diam, circular, flat, colony margin erose, surface white; reverse light yellow (2A5) in the centre, white at the margin, with pale yellow (2A3) pigmentation.

*Other examined isolates*: China, Guangdong Province, Shenzhen city, from marine algae *Ulva spinulosa*, May 2014, M.M. Wang (WL06823); ibid., WL06824.

*Notes*: The genus *Marquandomyces* Samson, Houbraken & Luangsa-ard was established in 2020, with *M. marquandii* (Massee) Samson, Houbraken & Luangsa-ard as the type species [47]. In this study, a new species of this genus was introduced, namely *M. ulvae*. Phylogenetically, this species was closed related to *M. damingensis* X.C. Wang, L.Y. Peng & W.Y. Zhuang and *M. tianshanicus* X.C. Wang, L.Y. Peng, Gafforov & W.Y. Zhuang (Figure 1), but differs in 57 bp and 63 bp in the LSU-ITS-tef1 dataset, respectively. Morphologically, *M. ulvae* was distinguished from the latter two species in the conidiogenous structures, and shape and size of conidia (conidiophores often reduced to phialides, conidia oval, 2.5–5 × 2–3.3 μm in *M. ulvae*, vs. conidiophores terverticillate or biverticillate, conidia ellipsoidal to fusiform, 3.0–4.0 × 2.5–3.0 µm in *M. damingensis*, and conidiophores terverticillate, conidia ellipsoidal to fusiform, 3.0–4.0 × 2.0–3.0 µm in *M. tianshanicus*) [48]. Otherwise, *M. ulvae* discovers from marine algae in China, differs from *M. damingensis* and *M. tianshanicus* from soil in China and Uzbekistan, respectively [48].

**Figure 5:**
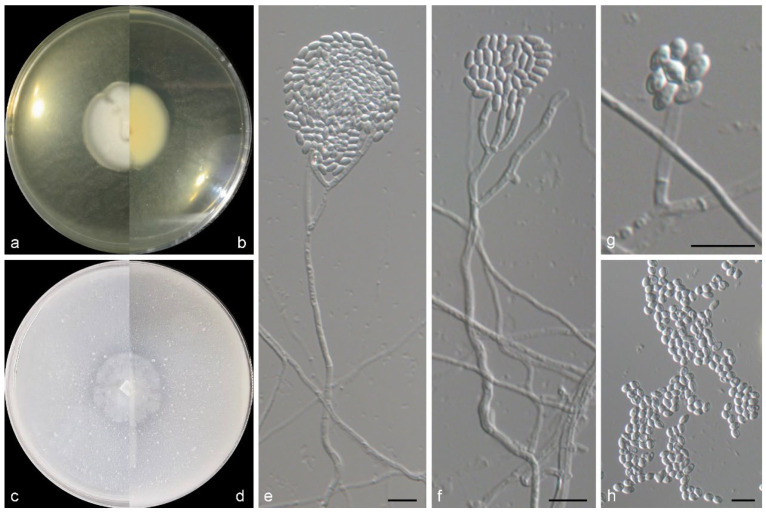
Morphological characters of *Stephanonectria arenicola* (from ex-type WL05612). (**a**–**d**) Colonies on PDA and OA after 7 d; (**e**–**g**) conidiophores and conidiogenous cells; (**h**) conidia in chains. Bars: (**e**–**h**) = 10 μm.

***Stephanonectria arenicola*** M.M. Wang & W. Li sp. nov., Figure 5.

*Classification: Ascomycota*, *Sordariomycetes*, *Hypocreales*, *Bionectriaceae*, *Stephanonectria*

*FungalNames*: FN 572363

*Etymology*: Epithet refers to the habitat of the type specimen, sand.

*Typus*: China, Shandong Province, Weihai city (122.19° E, 37.50° N), from intertidal sediment of a sand beach, November 2020, M.M. Wang and Y. Zheng (HMAS 353473, holotype designated here, dried culture on PDA; culture ex-type CGMCC 3.28726 = WL05612).

Mycelium consisting of branched, septate, hyaline, rough-, thin-walled hyphae, up to 3 μm wide. Conidiophores solitary or aggregate, (sub-)erect, arising from submerged and superficial hyphae, unbranched or branched, bearing 1–3 phialides per node, up to ca. 50 μm long, 2–3.5 μm wide at base, with 1–3 septa, hyaline, rough-walled, with cell walls usually thicker than those of vegetative hyphae. Phialides terminal or lateral, cylindrical or subulate, hyaline, thick-, smooth or slightly rough-walled, 9.5–30.5 × 2.3–3.5 μm; polyphialides not observed. Conidia aseptate, broad ellipsoid, occasionally with a slightly apiculate bases and rounded apices, hyaline, thin-, slightly rough-walled, 4–6.5 × 2.8–4.3 μm, forming conidial heads. Chlamydospores and sexual morph not observed.

*Culture characteristics*—Colonies on OA at 25 °C after 7 days reaching 10–12 diam, irregular, flat, colony margin undulate, surface white; reverse white. Colonies on PDA at 25 °C after 7 days reaching 10–15 mm diam, circular, raised, colony margin entire, surface white; reverse pale yellow (3A1) in the centre, white at the margin.

*Other examined isolates*: China, Shandong Province, Weihai city (122.19° E, 37.50° N), from intertidal sediment of a sand beach, November 2020, M.M. Wang and Y. Zheng (WL06829); ibid., WL06830.

*Notes*: The new species *Stephanonectria arenicola* is the fourth species of the genus *Stephanonectria* Schroers & Samuels after *S. chromolaenae* R.H. Perera & K.D. Hyde, *S. ellipsoidea* S.C. He, D.P. Wei & Jayaward., and *S. keithii* (Berk. & Broome) Schroers & Samuels. Morphologically, *S. arenicola* could be distinguished in the conidiogenous structures, and shape and size of conidia (conidiophores solitary or aggregate, unbranched or branched, bearing 1–3 phialides per node, conidia aseptate, broad ellipsoid, 4–6.5 × 2.8–4.3 μm in *S. arenicola*, vs. conidiophores sporodochial, densely arranged, irregularly branched, conidia ellipsoidal, 4.5–5.6 × 2–2.5 μm in *S. chromolaenae*, conidiophores sporodochial, irregularly branched, conidia oblong with obtuse ends, 6.2–6.8 × 3.3–3.7 μm in *S. ellipsoidea*, and conidiophores sporodochial, branched, condia ellipsoidal, 2.9–7.6 × 1.6–5.2 μm in *S. keithii*) [49,50,51]. Furthermore, the newly identified species exhibit distinct differences from the three other known species in habitats/hosts. *Stephanonectria arenicola* is found in intertidal sediment, while *S. chromolaenae* has been observed in the dead stem of *Chromolaena odorata*, *S. ellipsoidea* in dried fruit of a woody plant and *S. keithii* in the stalks of *Brassica* [49,50,51].

**Figure 6:**
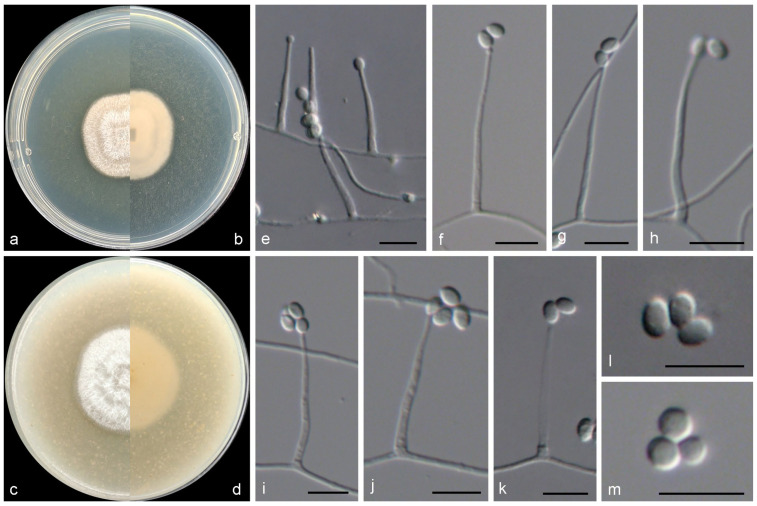
Morphological characters of *Verruciconidia oligospora* (from ex-type WL05964). (**a**–**d**) Colonies on PDA and OA after 7 d; (**e**–**k**) conidiophores and conidiogenous cells; (**l**,**m**) conidia. Bars: (**e**–**m**) = 10 μm.

***Verruciconidia oligospora*** M.M. Wang & W. Li sp. nov., Figure 6.

*Classification: Ascomycota*, *Sordariomycetes*, *Hypocreales*, *Bionectriaceae*, *Verruciconidia*

*FungalNames*: FN 572364

*Etymology*: Epithet refers to the less sporulation than other known species in the genus *Verruciconidia*.

*Typus*: China, Liaoning Province, Dalian city (122.99° E, 39.50° N), from intertidal sediment of a mud beach, May 2021, Y. Zheng and Z.H. Pan (HMAS 353474, holotype designated here, dried culture on PDA; culture ex-type CGMCC 3.28727 = WL05964).

Mycelium consisting of branched, septate, hyaline, smooth-, thin-walled hyphae, up to 3 μm wide. Conidiophores solitary, (sub-)erect, arising from submerged and superficial hyphae, unbranched, hyaline, slightly rough-walled, with cell walls usually thicker than those of vegetative hyphae. Phialides terminal or lateral, cylindrical or subulate, hyaline, thick, slightly rough-walled, 20.0–35.0 × 2.0–3.0 μm wide at base; polyphialides not observed. Conidia aseptate, ellipsoid to globose, hyaline, thin-, smooth-walled, 3.3–5.7 × 2.5–4.5 μm. Chlamydospores and sexual morph not observed.

*Culture characteristics*—Colonies on OA at 25 °C after 7 days reaching 15–20 diam, circular, crateriform, filamentous, colony margin entire, surface white; reverse white. Colonies on PDA at 25 °C after 7 days reaching 15–20 mm diam, circular, raised, filamentous, colony margin erose, surface white; reverse white.

*Other examined isolates*: China, Liaoning Province, Dalian city (122.99° E, 39.50° N), from intertidal sediment of a mud beach, May 2021, Y. Zheng and Z.H. Pan (WL06827); ibid., WL06828.

*Notes*: *Verruciconidia* L.W. Hou, L. Cai & Crous is a newly genus that established in 2023, with seven species including its type species *V. verruculosa* (W. Gams & Veenb.-Rijks) L.W. Hou, L. Cai & Crous [7]. In this study, the eighth species was introduced, namely *V. oligospora*. Phylogenetically, *V. oligospora* showed close relationship with *V. persicina* (Nicot) L.W. Hou, L. Cai & Crous and *V. verruculosa* (Figure 1), but differed by 61 bp and 99 bp in the LSU-ITS-tef1-rpb2 dataset, respectively. Morphologically, *V. oligospora* could be recognized in the shape and size of conidia (ellipsoid to globose, 3.3–5.7 × 2.5–4.5 μm in *V. oligospora*, vs. ellipsoid, ovoid, 4.2–6.2 × 2.4–3 μm in *V. persicina*, and ellipsoid, 4.5–6.2 × 2.5–3.5 μm in *V. verruculosa*) [7]. Additionally, the aforementioned species exhibit variations in their localities and hosts/habitats. For instance, *V. oligospora* from intertidal sediment in China, while *V. persicina* was discovered from coastal sand under *Ammophila arenaria* in France and *V. verruculosa* from agricultural soil in the Netherlands [7].

## 4. Discussion

The five new species described in this study were found to be phylogenetically well-located in five genera in three families in the order Hypocreales, with high supported values (BI = 0.97–1 and BS = 100; Figure 1). These include *Gliomastix* Guég., *Stephanonectria* and *Verruciconidia* in *Bionectriaceae*, *Marquandomyces* in *Clavicipitaceae*, and *Fusarium* Link in *Nectriaceae*, as determined by the LSU-ITS-tef1-rpb2 dataset (Figure 1). Morphologically, these species exhibited typical characteristics of *Acremonium* Link, yet differed from their closely related species in terms of conidiophores and shape and size of conidia, respectively. For example, all the known *Stephanonectria* species form sporodochial conidiophores [49,50,51] in contrast to the newly introduced species *S. arenicola* forms aerial conidiophores. *Verruciconidia oligospora* sp. nov. differs from its sister species in the size of conidia (3.3–5.7 × 2.5–4.5 μm in *V. oligospora*, vs. 4.2–6.2 × 2.4–3 μm in *V. persicina* and 4.5–6.2 × 2.5–3.5 μm in *V. verruculosa*) [7].

The hypocrealean fungi perform a variety of pivotal ecological functions within their respective environments, a fact that is exemplified by the five genera mentioned in this study. Members of the *Fusarium* (approximately 400 species) [52] are renowned pathogens of a wide range of plants and animals, and are frequently isolated from terrestrial and aquatic environments as documented in the FungalTraits database [18]. Species of *Gliomastix* (approximately 20 species) [52] are frequently documented as saprotrophic fungi [18] and are commonly found in soil, plants or air [7]. The genus *Marquandomyces* includes seven known species that have been extensively reported to play multiple crucial functions in agriculture and environmental remediation, for instance, as effective biocontrol agent against nematodes, notably reducing root galling in tomato and increasing the heat weights of lettuce [48]. The genus *Stephanonectria* was formerly considered to be saprotrophic fungi associated with litter, plants and soil [18]. The genus *Verruciconidia* has been found to be associated with air, plants and soil on a global scale [7]. The preponderance of evidence indicated that this group of fungi exhibits a wide geographical distribution and a robust capacity for environmental adaptation.

The broad ecological adaptability of hypocrealean fungi is attributable to their metabolic versatility, which concomitantly positions them as pivotal candidates for biotechnological innovation. The cross-sectoral utility of these fungi is evident in their capacity to fulfil specialised enzymatic and biochemical functions. A case in point is the mycoprotein synthesised by *F. venenatum* Nirenberg in the context of food manufacturing [53]. This mycoprotein is characterised by its high protein and cellulose content, and it has been commercialized as a sustainable meat alternative under the brand name Quorn [53]. In the field of chemical engineering, cutinase, a fungal enzyme produced by *F. oxysporum* Schltdl., has been shown to possess the capacity for polyethylene terephthalate modification [54]. Similarly, laccase-like enzymes derived from *G. murorum* (Corda) S. Hughes have demonstrated the ability to oxidise a wide range of organic compounds [55]. In the field of agriculture, *Beauveria bassiana* (Bals.-Criv.) Vuill. and *Lecanicillium lecanii* (Zimm.) Zare & W. Gams are effective entomopathogens that are capable of significantly reducing the survival and reproduction of insect pests like *Aphis gossypii* Glover through the mechanisms of direct infection and the production of toxic metabolites [56]. In the field of biomedicine, chlorinated orsellinic aldehydes derived from *A. sclerotigenum* (Moreau & R. Moreau ex Valenta) W. Gams have been shown to exhibit a high level of antifungal activity [57].

This study has expanded the corpus of knowledge concerning the ecological adaptation of the hypocrealean fungi. While seven *Fusarium* species have previously been reported from diverse host/habitat (mangrove algae, plants, sponges and sediments; etc.) in the marine environment with a wide geographic distribution and dominant presence [15,58,59] and *G. murorum* has been isolated from sediment of marine environments [60,61], members of the other three genera *Marquandomyces*, *Stephanonectria* and *Verruciconidia* were newly documented in marine environments. The present study lends further credence to the idea that hypocrealean fungi may be largely represented in marine environments. In the future, the study of these fungi will be pursued, including their species diversity, geographical distribution, host/substrate correlation and possible application value.

## Figures and Tables

**Table 1:** Strains examined in this study, with information on source, origin and GenBank accessions of the sequences.

Species	Strain Number and Status	Habitat/Host	Origin	GenBank Accession Number			
				ITS	LSU	tef1	rpb2
*Acremonium brachypenium*	CBS 866.73 T	*Cocos nucifera*	Sri Lanka	OQ429443	OQ055354	OQ470740	OQ453837
*A. brunneisporum*	CBS 413.76 T	*Colletotrichum dematium* on pod of *Albizzia lebbek*	India	OQ429444	OQ055355	OQ470741	OQ453838
*A. egyptiacum*	CBS 114785 T	Ground	Egypt	OQ429456	OQ055362	OQ470749	OQ453845
*Acrostalagmus luteo-albus*	CBS 112.16	--	UK	LR026668	LR025797	LR026369	LR026101
*Clonostachys grammicospora*	CBS 209.93 T	Trunk of dead standing tree	French Guiana	OQ910625	OQ910984	OQ944637	OQ927696
*C. spinulosispora*	CBS 133762 T	*Astrocarium* sp.	French Guiana	MH634702	KY006568	--	--
*C. vacuolata*	CBS 191.93 T	Bark	Venezuela, Edo	OQ910868	OQ911227	OQ944876	OQ927931
*Fusariella atrovirens*	CBS 311.73	Desert soil	Algeria	OQ429594	OR052105	OQ470904	OQ453993
*Fu. concinna*	CBS 312.73	Desert soil	Algeria	OQ429595	OQ055505	OQ470905	OQ453994
*Fu. curvata*	MFLUCC 15-0844 T	*Quercus* sp.	Thailand	KX025152	KX025154	KX025155	KX025156
*Fusarium acuminatum*	NRRL 36147 = CBS 109232	Man, bronchial secretion	--	GQ505452	GQ505452	GQ505420	GQ505484
*F. avenaceum*	NRRL 25128	*Hymenoptera ichneumonidae*	Poland	JF740894	JF740894	JF740751	JF741079
*F. celtidicola*	MFLUCC 16-0526 T	Dead branch of *Celtis australis*	Italy	MG873469	MG873466	MH576581	ON759296
*F. citricola*	CPC 27805 = CBS 142421 T	Crown of *Citrus reticulata*	Italy	LT746245	LT746245	LT746197	LT746310
*F. equiseti*	NRRL 26419 = BBA 68556 = CBS 307.94 T	Soil	Germany	NR_121457	--	GQ505599	GQ505777
*F. flavoides*	WL03768 = CGMCC 3.28711 T	Intertidal sediment	China, Shandong Province, Yantai city	**PV020684**	**PV020702**	**PV050414**	**PV023180**
	WL06821	Intertidal sediment	China, Shandong Province, Yantai city	**PV020685**	**PV020703**	**PV050415**	**PV023181**
	WL06822	Intertidal sediment	China, Shandong Province, Yantai city	**PV020686**	**PV020704**	**PV050416**	**PV023182**
*F. flocciferum*	NRRL 45999	--	United States CA	GQ505465	GQ505465	GQ505433	GQ505497
*F. iranicum*	CBS 143608 = CPC 30860 T	*Agaricus bisporus*	Orumieh-Salmas, Iran	LT970821	LT970821	LT970785	LT970757
*F. lateritium*	NRRL 13622	*Ulmus* sp.	USA	--	--	AY707173	JX171571
*F. napiforme*	CBS 748.97 T	*Pennisetum typhoides*	Namibia	KR071645	KU604071	AF160266	KU604233
*F. oxysporum*	CBS 144134 ET	*Solanum tuberosum*	Germany	--	--	MH485044	MH484953
*F. salinens*	CBS 142420 = CPC 26973 T	*Citrus sinensis* twigs	Italy	LT746241	LT746241	LT746193	LT746306
*F. thapsinum*	ATCC 200522 = CBS 777.96 = FRC M-6564 T	Stalk of *Sorghum* sp.	USA	MH862618	MH874241	MW928844	MW928833
	CBS 733.97 = NRRL 22045	--	--	KR071690	KU604054	AF160270	JX171600
*F. tricinctum*	CBS 393.93 ET	Winter wheat	Germany	HM068317	HM068317	AB674263	JX171629
*Gliomastix fasciculata*	WL05659 = CGMCC 3.28712 T	Intertidal sediment	China, Shandong Province, Weihai city	**PV020693**	**PV020711**	**PV050422**	**--**
	WL05858	Intertidal sediment	China, Shandong Province, Qingdao city	**PV020690**	**PV020708**	**--**	**--**
	WL05949	Intertidal sediment	China, Liaoning Province, Dalian city	**PV020691**	**PV020709**	**PV050420**	**PV023186**
	WL06005	Intertidal sediment	China, Liaoning Province, Huludao city	**PV020692**	**PV020710**	**PV050421**	**PV023187**
	WL06825	Intertidal sediment	China, Shandong Province, Weihai city	**PV020694**	**PV020712**	**PV050423**	**--**
	WL06826	Intertidal sediment	China, Shandong Province, Weihai city	**PV020695**	**PV020713**	**PV050424**	**--**
*G. masseei*	CBS 794.69 T	Dung of rabbit	Italy	OQ429601	OQ055510	OQ470911	OQ454000
*G. musae*	CBS 617.94 T	*Musa* sp.	Colombia	OQ429616	OQ055523	OQ470926	OQ454015
*G. murorum*	CBS 154.25 T	*Malus sylvestris*	--	OQ429613	HQ232063	OQ470923	OQ454012
*G. polychroma*	CBS 181.27 T	*Hevea brasiliensis*	Indonesia	OQ429629	OQ055528	OQ470931	OQ454020
*G. roseogrisea*	CBS 134.56 T	Grassland soil	India	OQ429639	OQ055545	OQ470948	OQ454037
*G. tumulicola*	CBS 127532 T	White salt-like masses on teh central part of teh painting	Japan	OQ429641	OQ055547	OQ470950	OQ454039
*Hydropisphaera fungicola*	A.R. 4170 T	*Ulocladium atrum*	USA	OQ429666	OR052107	OQ470973	OQ454063
*H. peziza*	CLL 14063	Dead wood	France	OQ429667	OQ055572	OQ470974	OQ454064
*H. suffulta*	CBS 122.87	*Cocos nucifera*	Indonesia	OQ429672	OQ055577	OQ470979	OQ454068
*Marquandomyces damingensis*	JJJ73-30 = CGMCC 3.28567 T	Soil	China, Hebei Province	PQ484187	PQ484201	PQ469018	--
*M. marquandii*	CBS 182.27	Soil	USA	MH854923	MH866418	EF468793	EF468942
*M. sinensis*	CGMCC 3.25518 T	Soil	China, Guizhou Province, Guiyang city	OR680543	OR680610	OR858937	OR842958
*M. tashkentensis*	UZ13-25 = CGMCC 3.28568 T	Soil	Uzbekistan, Tashkent	PQ484189	PQ484203	PQ469020	--
*M. tianshanicus*	UZ14-25 = CGMCC 3.28569 T	Soil	Uzbekistan, Tashkent	PQ484190	PQ484204	PQ469021	--
*M. ulvae*	WL01242 = CGMCC 3.28716 T	*Ulva spinulosa*	China, Guangdong Province, Shenzhen city	**PV020687**	**PV020705**	**PV050417**	**PV023183**
	WL06823	*Ulva spinulosa*	China, Guangdong Province, Shenzhen city	**PV020688**	**PV020706**	**PV050418**	**PV023184**
	WL06824	*Ulva spinulosa*	China, Guangdong Province, Shenzhen city	**PV020689**	**PV020707**	**PV050419**	**PV023185**
*M. uzbekistanicus*	UZ11-45 = CGMCC 3.28570 T	Soil	Uzbekistan, Tashkent	PQ484192	PQ484206	PQ469023	--
*M. yaoyijianii*	HLJ55-10 = CGMCC 3.28571 T	Soil	China, Heilongjiang Province	PQ484193	PQ484207	PQ469024	--
*Nectria cinnabarina*	CBS 125165 T	*Aesculus* sp.	France	HM484548	HM484562	HM484527	KM232402
*N. nigrescens*	CBS 125148 T	Wood	USA	HM484707	HM484720	HM484672	KM232403
*N. polythalama*	CBS 128672	Decaying twig	New Zealand	OQ429722	OQ055623	OQ471033	OQ454123
*Neocosmospora falciformis*	CBS 475.67 = IMI 268681 T	Human mycetoma	Puerto Rico	MG189935	MG189915	LT906669	LT960558
*Ne. haematococca*	CBS 119600 ET	Dying tree	Sri Lanka	KM231797	KM231664	KM231926	LT960561
*Paragliomastix luzulae*	CBS 935.69	*Fagus sylvatica*	Germany	OQ429774	OQ055672	OQ471102	OQ454185
*P. rosea*	CBS 277.80A T	--	India	OQ429775	OQ055673	OQ471103	OQ454186
*P. znieffensis*	CBS 140584 T	*Cyathea* sp.	Martinique	OQ429776	KU198185	OQ471104	OQ454187
*Pseudosynnemellisia favida*	CGMCC 3.22477 = LC15930 T	Sediment	China, Guangdong Province, Shenzhen city	OQ798973	OQ758164	OQ809058	OQ809024
	CGMCC 3.22483 = LC15931	Sediment	China, Guangdong Province, Shenzhen city	OQ798974	OQ758165	OQ809059	OQ809025
*Scolecofusarium ciliatum*	CBS 144385	Leaf of *Fagus sylvatica*	Belgium	KJ125591	KJ126479	MW834297	KP835472
	CBS 191.65 NT	*Fagus sylvatica*, branch canker	Germany	MW827636	MW827677	MW834296	MW834035
*Sesquicillium rossmaniae*	CBS 211.93 T	Twigs of recently dead tree	French Guiana, Piste de Saint-Elie	OQ911299	OQ911360	OQ944534	OQ914852
*Se. saulense*	BRFM 2782 T	Dead bark of *Bauhinia* sp.	French Guana, Saül	MK635054	--	--	--
*Se. sesquicillii*	CBS 180.88 T	Twigs and lichen	Guyana, Cuyuni-Mazaruni Region	OQ911300	MH873818	OQ944535	--
*Stephanonectria arenicola*	WL05612 = CGMCC 3.28726 T	Intertidal sediment	China, Shandong Province, Weihai city	**PV020699**	**PV020717**	**PV050428**	**--**
	WL06829	Intertidal sediment	China, Shandong Province, Weihai city	**PV020700**	**PV020718**	**PV050429**	**--**
	WL06830	Intertidal sediment	China, Shandong Province, Weihai city	**PV020701**	**PV020719**	**PV050430**	**--**
*S. chromolaenae*	MFLUCC 18-0589 T	Dead stem of *Chromolaena odorata*	Thailand, Chiang Mai Province	NR_189387	NG_241983	--	--
*S. ellipsoidea*	KUNCC 22-12394 T	Dried fruit of a woody plant	China, Yunnan Province, Kunming City	OP205363	OP205375	--	--
*S. keithii*	CBS 100007	*Beilschmiedia tawa*	New Zealand	OQ429871	OQ430120	OQ471202	OQ454269
*Verruciconidia erythroxyli*	CBS 728.87 T	*Erythroxylum areolatum*	Cuba	OQ429910	OQ430161	OQ471240	OQ454307
*V. infuscata*	CBS 100888 T	Air	Japan	OQ429911	OQ430162	OQ471241	OQ454308
*V. oligospora*	WL05964 = CGMCC 3.28727 T	Intertidal sediment	China, Liaoning Province, Dalian city	**PV020696**	**PV020714**	**PV050425**	**PV023188**
	WL06827	Intertidal sediment	China, Liaoning Province, Dalian city	**PV020697**	**PV020715**	**PV050426**	**PV023189**
	WL06828	Intertidal sediment	China, Liaoning Province, Dalian city	**PV020698**	**PV020716**	**PV050427**	**PV023190**
*V. persicina*	CBS 310.59 T	Coastal sand under *Ammophila arenaria*	France	OQ429921	OQ430172	OQ471251	OQ454318
*V. quercina*	CBS 469.67 T	*Quercus* sp.	--	OQ429925	OQ430176	OQ471255	OQ454322
*V. siccicapita*	CBS 378.70A T	Soil	Thailand	OQ429928	OQ430179	OQ471258	OQ454325
*V. unguis*	CBS 424.93 T	Nail of man	Netherlands	OQ429929	OQ430180	OQ471259	OQ454326
*V. verruculosa*	CBS 989.69 T	Agricultural soil	Netherlands	OQ429933	OQ430184	OQ471263	OQ454330

Notes: T = ex-type, ET = epitype, NT = neotype. Sequences newly generated in this study are marked in bold.

## Data Availability

All sequences generated in this study (PV020684-PV020719, PV023180-PV023190, and PV050414-PV050430) were submitted to GenBank (https://www.ncbi.nlm.nih.gov, accessed on 15 January 2025).

## Supplementary Materials

The following supporting information can be downloaded at: https://www.mdpi.com/article/10.3390/jof11070476/s1, Figure S1: Fifty percent majority rule consensus tree from a Bayesian analysis based on three-locus combined datasets (ITS-tef1-rpb2) showing the phylogenetic relationships of *Fusarium* (a), *Gliomastix* (b), *Marquandomyces* (c), *Stephanonectria* (d) and *Verruciconidia* (e). The Bayesian posterior probabilities (PP > 0.9) and PhyML bootstrap support values (BS > 50%) are displayed at the nodes (PP/BS). Ex-type cultures are indicated with “T”, epi-type with “ET” and neo-type with “NT”. New species introduced in this paper are marked in bold.
