# Supplementary figures and images for: Five New Hypocrealean Species from Algae and Sediment in the Intertidal Zones of China
Source: J Fungi (Basel). 2025 Jun 23;11(7):476. doi: 10.3390/jof11070476 (PMC12295223; doi:10.3390/jof11070476)

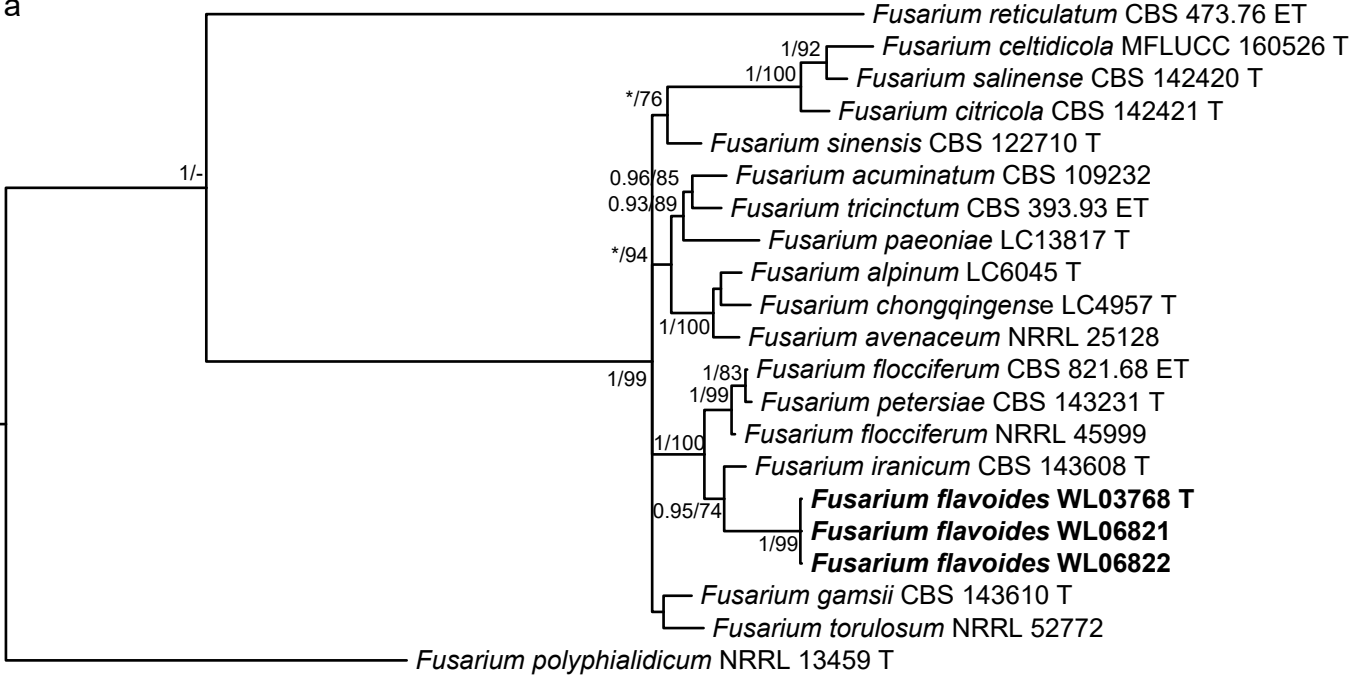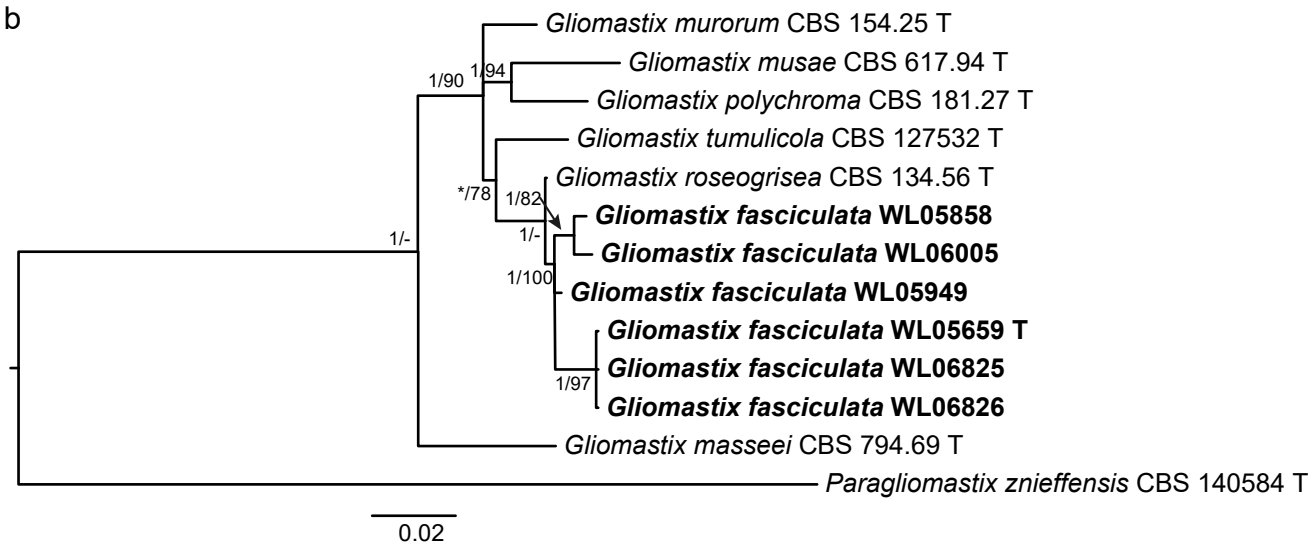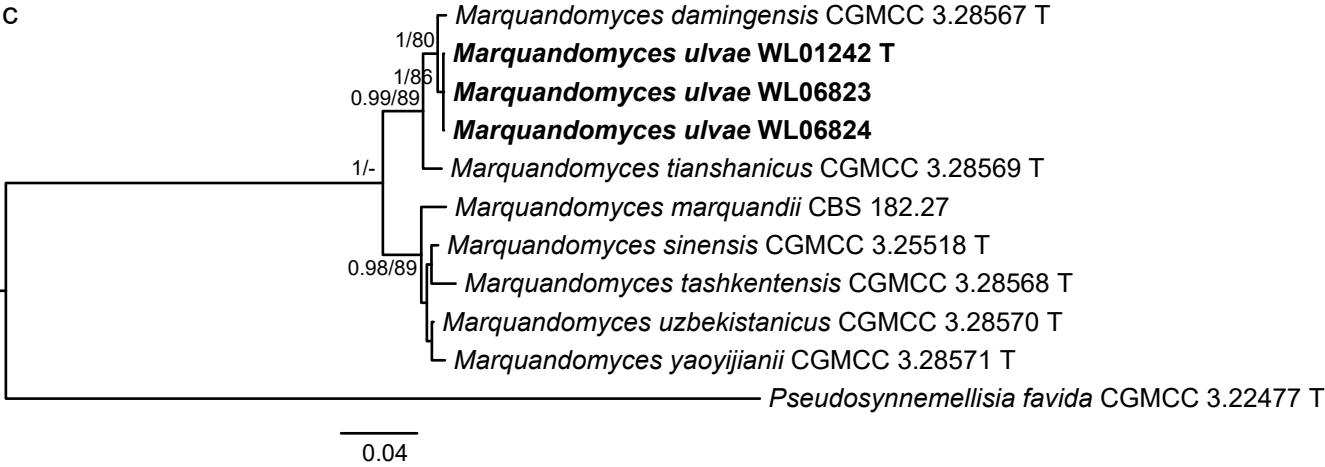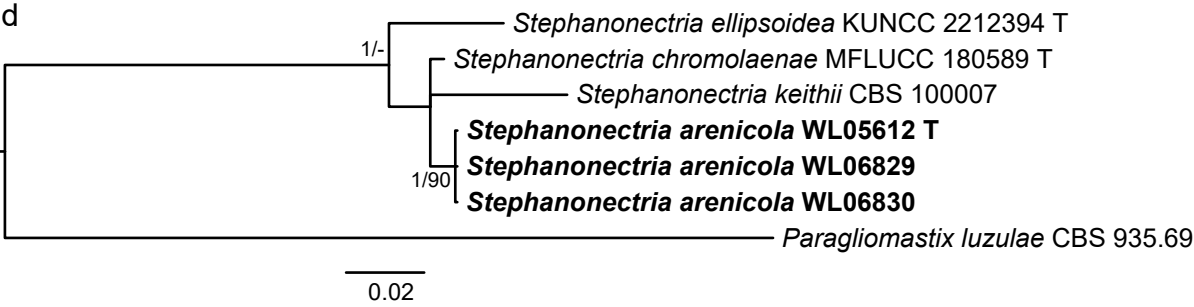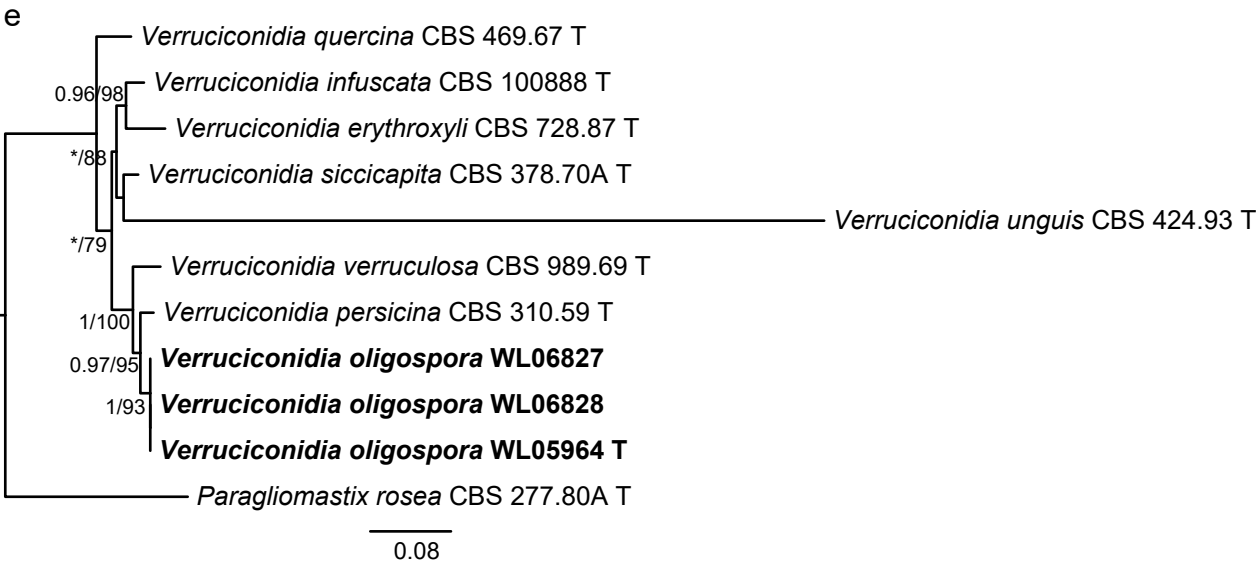

Supplement: Supplementary file 1 [file jof-11-00476-s001.zip › jof-3486468-supplementary.pdf]
